# Doxorubicin resistant choriocarcinoma cell line derived spheroidal cells exhibit stem cell markers but reduced invasion

**DOI:** 10.1007/s13205-022-03243-x

**Published:** 2022-07-20

**Authors:** Reham M. Balahmar, Venkataraman Deepak, Shiva Sivasubramaniam

**Affiliations:** 1 School of Science and Technology, Nottingham Trent University, Clifton Lane, Nottingham, NG11 8NS UK; 2 School of Human Sciences, College of Life and Natural Sciences, University of Derby, Kedleston Road, Derby, DE22 1GB UK

**Keywords:** Trophoblast, Stem-like cells, Doxorubicin, Spheroids, Choriocarcinoma cell lines

## Abstract

Cell cycle-specific cancer chemotherapy is based on the ability of a drug to halt, minimise or destroy rapidly dividing cells. However, their efficacy is limited by the emergence of a self-renewing cell pool called “cancer stem cells” (CSC). Choriocarcinoma is a tumour of trophoblastic tissue. We, in this study, analysed whether spheroids generated from doxorubicin-treated and non-treated choriocarcinoma cell lines exhibit markers of stem cells. Two choriocarcinoma cell lines, namely JEG-3 and BeWo, were used in this study. Spheroids were generated from doxorubicin-treated cells and the non-treated cells under non-adherent condition, followed by analysis of stem-cell markers’ expression, namely *NANOG, OCT4* and *SOX2*. Immunofluorescence analysis suggested a general increase in the markers’ concentration in spheroids relative to the parental cells. RT-qPCR and immunoblots showed an increase in the stem-cell marker expression in spheroids generated from doxorubicin-treated when compared to non-treated cells. In spheroids, *Sox2* was significantly upregulated in doxorubicin-treated spheroids, whereas *Nanog* and *Oct4* were generally downregulated when compared to non-treated spheroids. Both 2D and 3D invasion assays showed that the spheroids treated with doxorubicin exhibited reduced invasion. Our data suggest that choriocarcinoma cell lines may have the potential to produce spheroidal cells, yet the drug-treatment affected the invasion potential of spheroids.

**Supplementary Information:**

The online version contains supplementary material available at 10.1007/s13205-022-03243-x.

## Introduction

Several similarities have been shown between tumour invasion and trophoblast development. They both have the capacity to proliferate, differentiate and invade through the surrounding tissues by degrading the extracellular matrix (ECM), evading the immune response and establishing nutrient supply by angiogenesis (Ferretti et al. 2007; Lorena et al. 2021). However, the invasion of trophoblast cells into the uterine wall is a unique and controlled process, which is essential for foetal development (Louwen et al. 2012; Huppertz 2018). In gestational trophoblastic diseases such as choriocarcinoma, the trophoblast cells can become tumorigenic. In fact, choriocarcinoma is a malignant tumour of the uterus which originates in the foetal chorion. It is characterized by abnormal proliferation of trophoblast without the formation of chorionic villi (Greaves and Maley 2012; Kim et al. 2019). This gestational cancer is chemotherapy-sensitive but metastatic (Braga et al. 2019).

Chemotherapeutic drugs for cancer that target rapidly dividing cells are limited by their inability to differentiate between the physiologically rapidly dividing host cells and the cancer cells, the latter often results in the development of a self-renewing pool of resistant cells called cancer stem cells (CSCs) (Phi et al. 2018)). This small pool of cancer cells are resistant to the chemotherapeutic drugs. They contribute to recurrence and aggravation of cancers (Cho and Kim 2020). CSCs or stem-like cells are reported to be enriched in tumour-derived spheroids (Ishiguro et al. 2017). Tumour spheroids can be formed as a 3D in vitro culture system which can mimic the tumour microenvironment. This system has an added advantage of histologically mimicking in vivo tumour with tumour cell heterogeneity and ability to grow into tumours when mechanically dissociated (Wei et al. 2012; Ishiguro et al. 2017; Schultz et al. 2017). CSCs essentially express factors like *SOX2*, *OCT4* and *NANOG* called stemness factors (Liu et al. 2013; Basati et al. 2020). *SOX2* is a transcription factor belonging to the SOX B1 group of genes in the SOX (SRY related HMG box) family (Feng and Wen 2015). *SOX2* expression has been implicated in various cancer stem cells. It has been reported to be involved in preservation of the development potential of a stem cell (Avilion et al. 2003), *OCT4* is a member of the POSU family of transcription factors and an important pluripotency regulator. It is found to be essential for the formation of naïve epiblast and when the *OCT4* genes are silenced in embryonic stems cells they lose their pluripotency and start differentiating and resulting in trophoblast like morphology (Hough et al. 2006; Radzisheuskaya and Silva 2014). *NANOG* is a homeobox containing a transcription factor which is involved in the maintenance of pluripotency in inner cell mass and in the undifferentiated embryonic stem cell propagation (Chambers et al. 2003; Rodda et al. 2005). The expression of these stemness markers in several cases positively correlates with the tumour progression and poor prognosis (You et al. 2017; Yang et al. 2018; Basati et al. 2020). In this study, we developed doxorubicin resistant spheroids from JEG-3 and BeWo cells in vitro, and explored their invasive potential both with and without pre-treating them with doxorubicin. We also compared the expressions of stemness factors such as *NANOG*, *OCT4* and *SOX2* in the parental and spheroidal cells (untreated and doxorubicin-treated) generated from the cell lines.

## Materials and methods

### Cell lines culture

JEG-3 was cultured in EMEM medium (Lonza, UK), whereas BeWo was cultured in Ham’s F12 (Lonza, UK). Both media were supplemented with 10% (v/v) FBS (foetal bovine serum—FBS; Lonza, UK), 1% (v/v) penicillin/streptomycin (Lonza, UK), and 1% (w/v) L-glutamine (Lonza, UK). Cells were incubated at 37 °C in a humidified atmosphere of air containing 5% (v/v) CO_2_.

### Generation of non-resistant and drug-resistant (Dox-resistant) spheroids

Spheroids were produced as reported previously (Balahmar et al. 2018). In brief, spheroid cells were grown under normal conditions for 72 h. After 72 h, cells were trypsinized and were seeded (5 × 10^6^ cells/per flask) in an ultra-low attachment flask for 3–5 days. Spheroids were separated from single cells through gravity separation for 10 min and cultured as required. The same methodology was followed for producing treated spheroids with 250 ng/mL of doxorubicin. The expression of *NANOG*, *OCT4* and *SOX2* was later investigated in both untreated and treated spheroids using immunofluorescence, RT-qPCR, immunoblotting, 2D and 3D invasion assays.

### Immunofluorescence

Spheroids (non-treated and doxorubicin**-**resistant) were collected and centrifuged at 500 × *g* for 10 min, after washing with 1X PBS (Sigma-Aldrich, UK). The collected spheroids were fixed in 4% formaldehyde for 30 min at room temperature and washed three times with 1X PBS. Nonspecific reactivity was blocked by incubating the cells in a blocking solution (1% BSA in PBS Tween20) (Sigma, UK) for one hour. Spheroids were incubated overnight at 4 °C with respective primary antibodies anti-*Nanog* (2 mg/ml; 1:50, OAAB11202 Aviva Systems Biology, San Diego, USA), anti-*Oct4* (0.5 mg/mL; 1:200, ab18976 Abcam, Cambridge, UK) and anti-*Sox2* (1 mg/mL; 1:500, ab97959 Abcam, Cambridge, UK). This was followed by the treatment with secondary antibody. After washing Spheroids with PBS transferred onto slides and mounted with two drops of VECTASHIELD^®^ HardSet^™^ mounting medium, the boundaries were secured by means of a hydrophobic image barrier pen (Vector Laboratories, Burlingame, USA). Parental cells were grown onto sterile glass poly-l-lysine coated coverslips GG-18-PLL (neuVitro, USA, Germany), under the respective growth conditions.

### RNA extractions and quantitative real-time PCR (qRT-PCR)

qRT-PCR was carried out to determine the mRNA expression of each marker of interest. RNeasy^®^ Plus Mini-kit (Qiagen, Germany) was used to extract the total RNA from the harvested cells (both parental and spheroidal of JEG-3 and BeWo) according to manufacturer’s instructions. cDNA was then synthesized from RNA extracted using SuperScript™ II Reverse Transcriptase kit (Thermo Fisher (Invitrogen®), USA) according to manufacturer’s instructions. The primers were designed using the Primer 3^®^ Input version 4 software. Details of primers (supplied by MWG Eurofin, Germany). The qRT-PCR was performed using the Rotor-Gene 6000 real-time PCR cycler (Qiagen, Germany) using SYBR green method. The mRNA expression of each target factors was normalised against averaged expression of the housekeeping genes [hypoxanthine phosphoribosyltransferase-1 (HPRT1) and TATA Box Binding Protein (TBP1)] in the same samples and 2^−∆∆ Ct^ method was used for calculation.

### Immunoblotting

The protein extraction of harvested cells was measured by the BCA protein assay according to the manufacturer’s protocol (Pierce, Rockford, USA). Cells were harvested with 300 μL RIPA buffer (Sigma-Aldrich, UK) with 0.2 mL protease inhibitor cocktail (Roche, UK), 0.2% (v/v) and 1 mM Na3VO4 (Sigma-Aldrich, UK) and the resulting cell lysate was boiled for 5 min at 95 °C followed by storing the lysate − 20 °C and used whenever needed. Protein samples (50 μg) were separated by SDS/PAGE in 10% or 12% polyacrylamide gel using a Bio-Rad Mini-Protean III system (Hercules, California, USA) which were then transferred to nitrocellulose membranes in a Bio-Rad Trans-Blot system; using electro-transfer buffer, membranes were blocked using 3% w/v BSA in 1X TBS-Tween20. Membranes were incubated overnight at 4 °C with respective primary antibodies anti-*Nanog* (2 mg/ml; 1:500, OAAB11202 Aviva Systems Biology, San Diego, US), anti-*Oct4* (0.5 mg/ml; 1:500, ab18976 Abcam, Cambridge, UK) and anti-*Sox2* (1 mg/ml; 1:1000, ab97959 Abcam, Cambridge, UK), followed by incubation with respective secondary antibodies (1:10,000, Sigma) for 1 h at room temperature. The Advanced Image Data Analysis Software (Fuji; version 3.52) was used for densitometry quantified of western blot analysis. Qualitative analysis was normalized in relation to β-actin expression.

### Cell invasion assay (2D)

Cell invasion assay was carried out using the BD Falcon^™^ BioCoat tumour invasion systems (Corning, Arizona, USA) with fluoroBlock^™^ 96 well insert plate according to manufacturer’s guidelines. This was also compared with migration of cells through uncoated BD Falcon™ FluoroBlok^™^ 96 well insert plates (see Balahmar et al. 2018) for details). In this study, spheroid cells were used that have a total diameter above 8.0 μm. Therefore, the spheroids were disintegrated by using trypsin into single cells before performing the invasion assay. The number of cells invaded from the tumour invasion plate together with percentage invasion (see below for equation) was analysed using Image J software.

$$ {\text{Invasion}} \% = \frac{{{\text{Number of cells invaded}} }}{{\text{Number of cells migrated}}} \times 100. $$

### Cultrex^®^ 96 well 3D spheroid basement membrane extract (BME) cell invasion assay

The monolayer cell invasion systems are commonly used to evaluate invasion of single cells. Therefore, this method in this research was not sufficient to study the invasion potential of spheroidal cells. Although the spheroids were artificially separated into single cells to suit the 2D invasion assay, it is not entirely appropriate to compare the invasive behaviour of untreated and doxorubicin-treated spheroids. Therefore, the 3D invasion assay was carried out according to the manufacturer’s guidelines (Amsbio, UK). All confocal images were analysed by Image J software to measure changes in the invasion area at 12, 24 and 48 h.

### Statistical analyses

All data are presented as means ± SEM. Statistical significance was determined using a *t* test, a one-way ANOVA followed by Tukey’s and two-way ANOVA followed by Sidak’s test for multiple comparisons and for *t* tests *p* < 0.05 was considered statistically significant. (GraphPad Prism, version 6, California, USA).

## Results

### Generation of (non-resistant and doxorubicin-resistant) spheroids

**Fig. 1:**
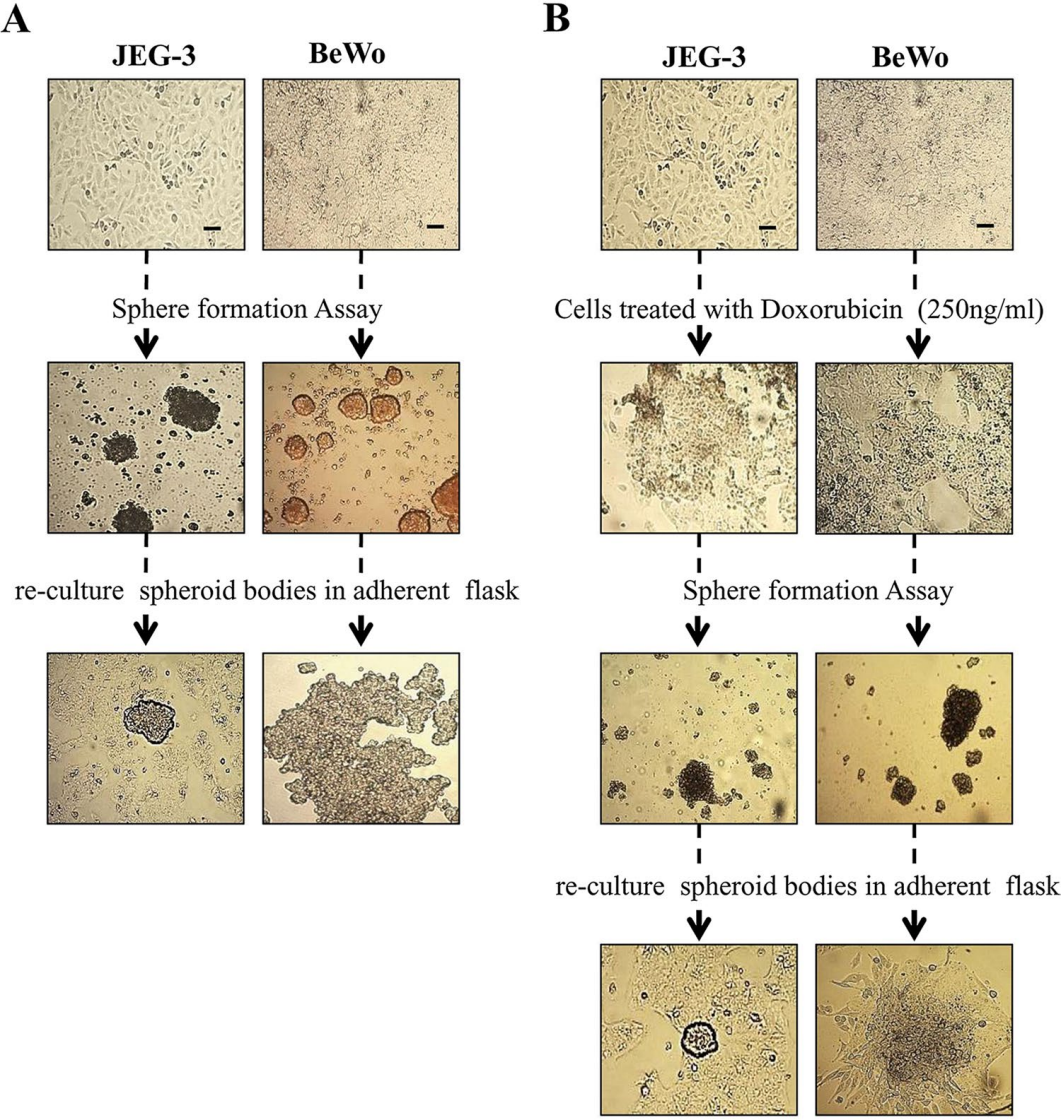
Generation of non-resistant and doxorubicin-resistant spheroids from choriocarcinoma cell lines. The ability of JEG-3 and BeWo cells to generate non-resistant spheroids is shown in Panels **A**. Row 1 = Parental cells without treatment; Row 2 = Spheroidal cells produced from non-adherent 3D culture; Row 3 = the ability of spheroidal cells to regrow onto normal adherent 2D culture. Likewise, JEG-3 and BeWo have shown the ability to generate doxorubicin-resistant spheroids shown in Panel **B**. Row 1 = Parental cells without treatment; Row 2 = Parental cells treated with 250 ng/ml of doxorubicin. Row 3 = Spheroidal cells produced from non-adherent 3D culture; Row 4 = the ability of spheroidal cells treated to re-grow onto normal adherent 2D culture. Scale bar = 100 μm

Both the cell lines were able to generate spheroids with and without the treatment of doxorubicin. To verify that spheroids (non-resistant and doxorubicin-resistant) are not just an aggregate of cells, we proceeded to disaggregate and split the spheroids into single cells by gravity separation and cultured them again under adherent conditions to assess their continued ability to self-grow. These cells attached and started to grow under normal conditions (Fig. 1).

### Expression of Nanog, Oct4 and Sox2 in choriocarcinoma cell lines

**Fig. 2:**
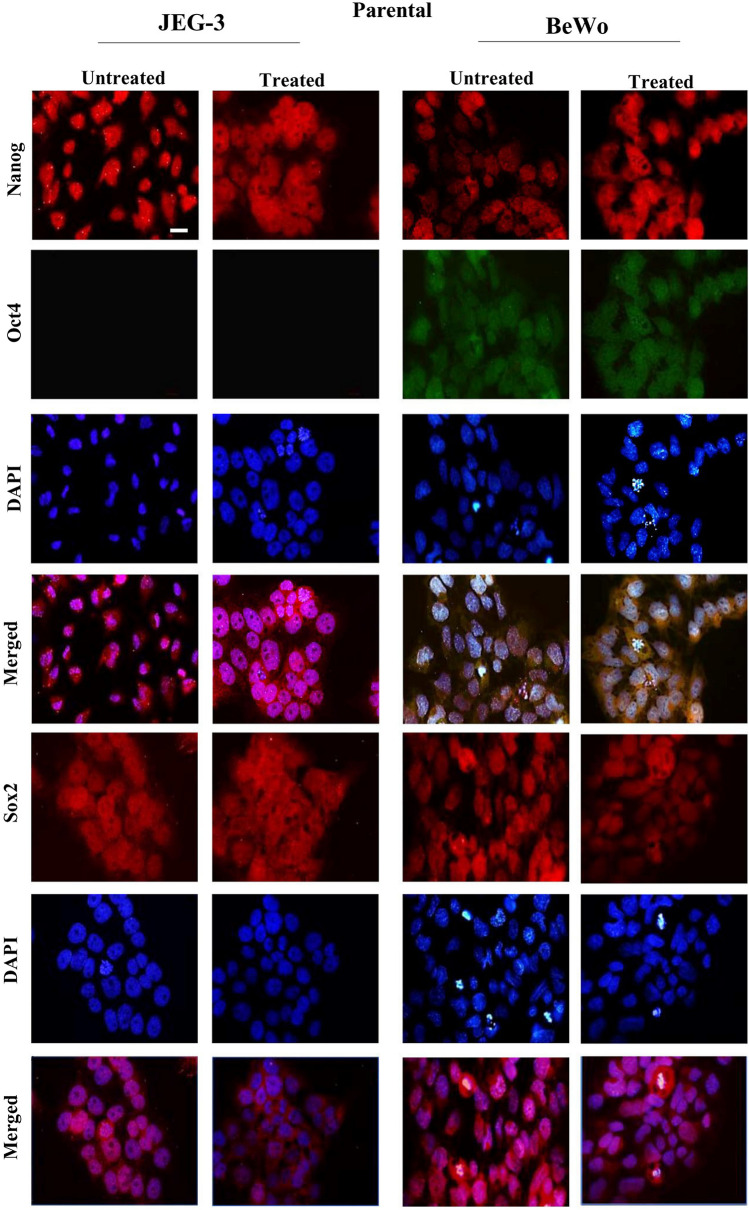
Expression of Nanog, Oct4 and Sox2 in placenta choriocarcinoma parental cells. Immunofluorescence photo-micrographs of Nanog, Oct4, Sox2 and with DAPI staining in choriocarcinoma parental cell lines of JEG3 and BeWo are shown in the left and the right panels, respectively. Note JEG-3 cells are not showing any immunoreactivity for Oct4. Objective magnification 20X. Scale bar = 50 μm

**Fig. 3:**
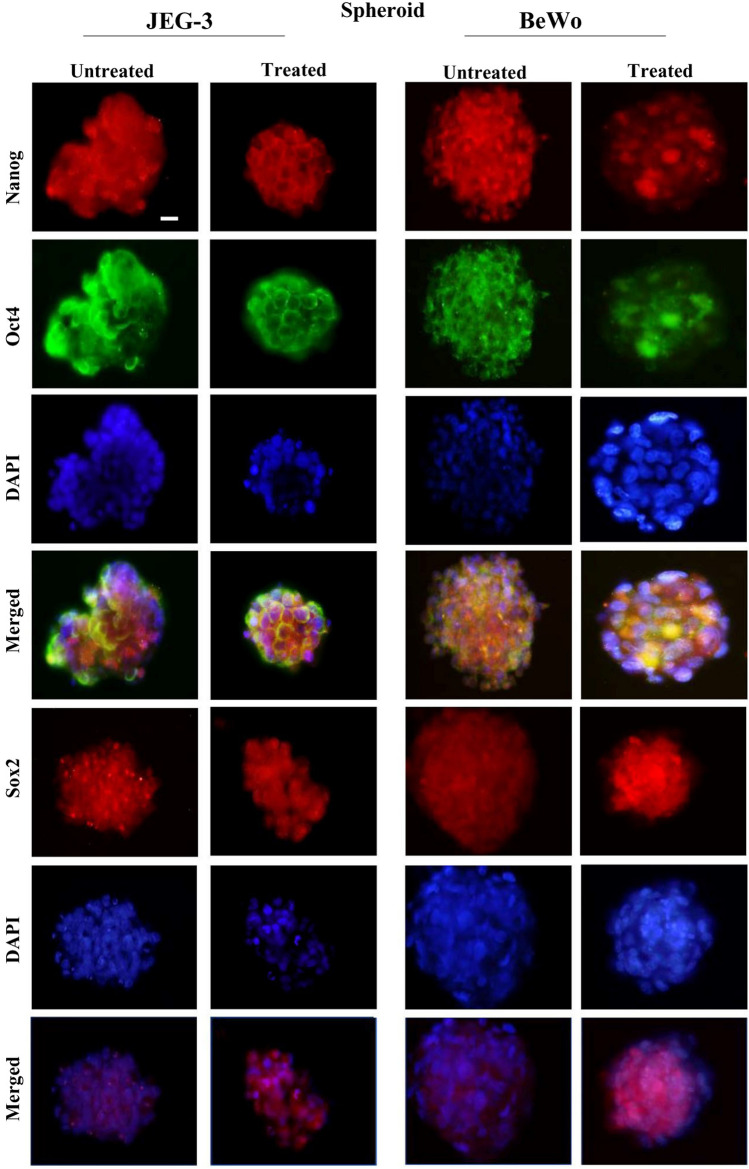
Expression of Nanog, Oct4 and Sox2 in placental choriocarcinoma spheroids. Immunofluorescence micrographs of Nanog, Oct4, Sox2 and with DAPI staining in spheroidal cells generated from choriocarcinoma cell lines (JEG-3and BeWo) are shown in the left and the right panels, respectively. The spheroidal cells of both choriocarcinoma cells are shown positive immunoreactivity for all stem cell markers. Objective magnification 20X. Scale bar = 50 μm

The presence of the stem cells was checked by studying the expression of stem-cell markers such as *Nanog*, *Oct4* and *Sox2*. Spheroids that were generated from both cell lines showed positive staining for *Nanog*, *Oct4* and *Sox2* (Fig. 2B). In the case of BeWo, the parental cells (both non-resistant and doxorubicin-resistant) expressed *Nanog* and *Sox2*, whereas the expression of *Oct4* was not observed in JEG-3 parental cells (both non-resistant and doxorubicin-resistant) (Fig. 2A). Yet the staining of *Nanog* and *Sox2* was consistent in JEG-3 parental and spheroidal cells. The immunofluorescence *Nanog, Oct4, Sox2* of the spheroidal cells (both non-resistant and doxorubicin-resistant) generated from JEG3 and BeWo are shown in Fig. 3. Interestingly, both doxorubicin-treated and untreated spheroidal cells showed immunoreactivities for all three stem cell markers.

We further examined the expressions of these factors by qRT-PCR (mRNA) and immunoblotting (protein) in the parental cells and spheroids. The data showed that *SOX2, OCT4* and *NANOG* exhibited a differential expression of mRNA patterns in parental and spheroidal cells (both untreated and treated). (Image provided as a supplement).

**Fig. 4:**
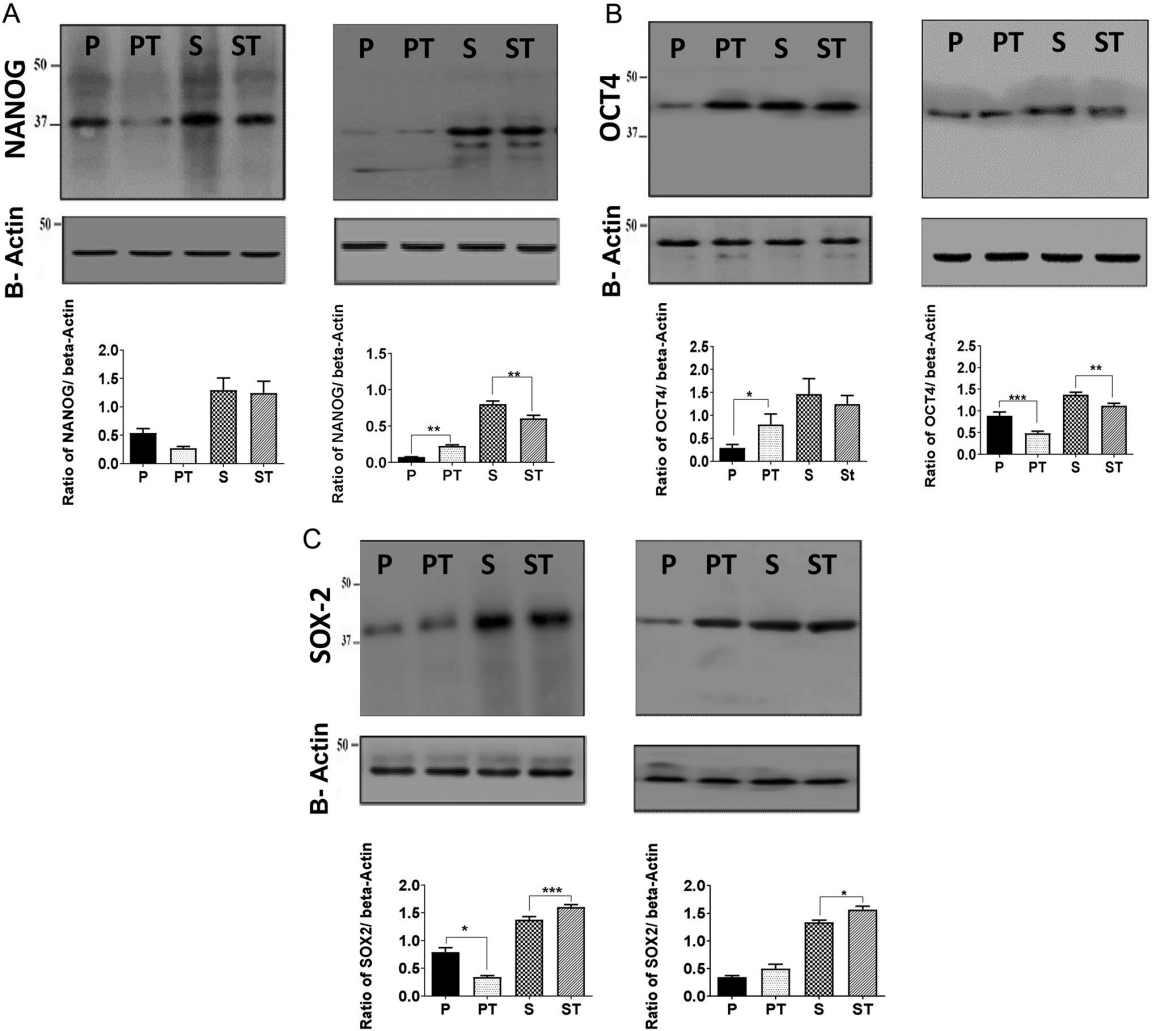
Immunoblot of Nanog, Oct4 and Sox2 expression in choriocarcinoma cells. Immunoblots showing the expression of Nanog, Oct4 and Sox2 in panels **A** to **C** (left panels JEG-3 and BeWo right panels). Corresponding β-actin loading controls in JEG-3and BeWo untreated and treated choriocarcinoma parental cells and spheroids. Data represent mean ± SEM of three individual experiments, each performed in triplicate (***p < 0.001;**p < 0.01,*p < 0.05); *P* Parental; *PT* Parental treated; *S* Spheroid; *ST* Spheroid treated

Further comparative analysis of their protein expressions is given in Fig. 4. Despite the individual differences, the expressions of the stem cell markers in spheroidal cells (both untreated and doxorubicin-treated) were higher than that of parental counterparts. In parental cells, treatment of doxorubicin had differential effect on the stem cell markers of the two cell lines. *Nanog* was slightly reduced (non-significant) in JEG-3, whereas it is significantly increased in the doxorubicin treated parental BeWo cells. Similarly, *Oct4* was significantly increased in the parental treated JEG-3 cells, whereas its expression was significantly reduced in BeWo cells. *Sox2* expression in significantly reduced in JEG-3 parental treated cells, whereas no significant difference was observed in BeWo cells. In spheroids, there was no significant difference in the expression of *Nanog* and *oct4* in JEG-3 doxorubicin-treated spheroids, whereas both the proteins were significantly reduced in BeWo cells. Interestingly, the doxorubicin-treated spheroids of both JEG-3 and BeWo cells showed a significant increase in *Sox2* expressions when compared with the non-treated spheroids.

### The invasion ability of spheroids from choriocarcinoma cell lines

**Fig. 5:**
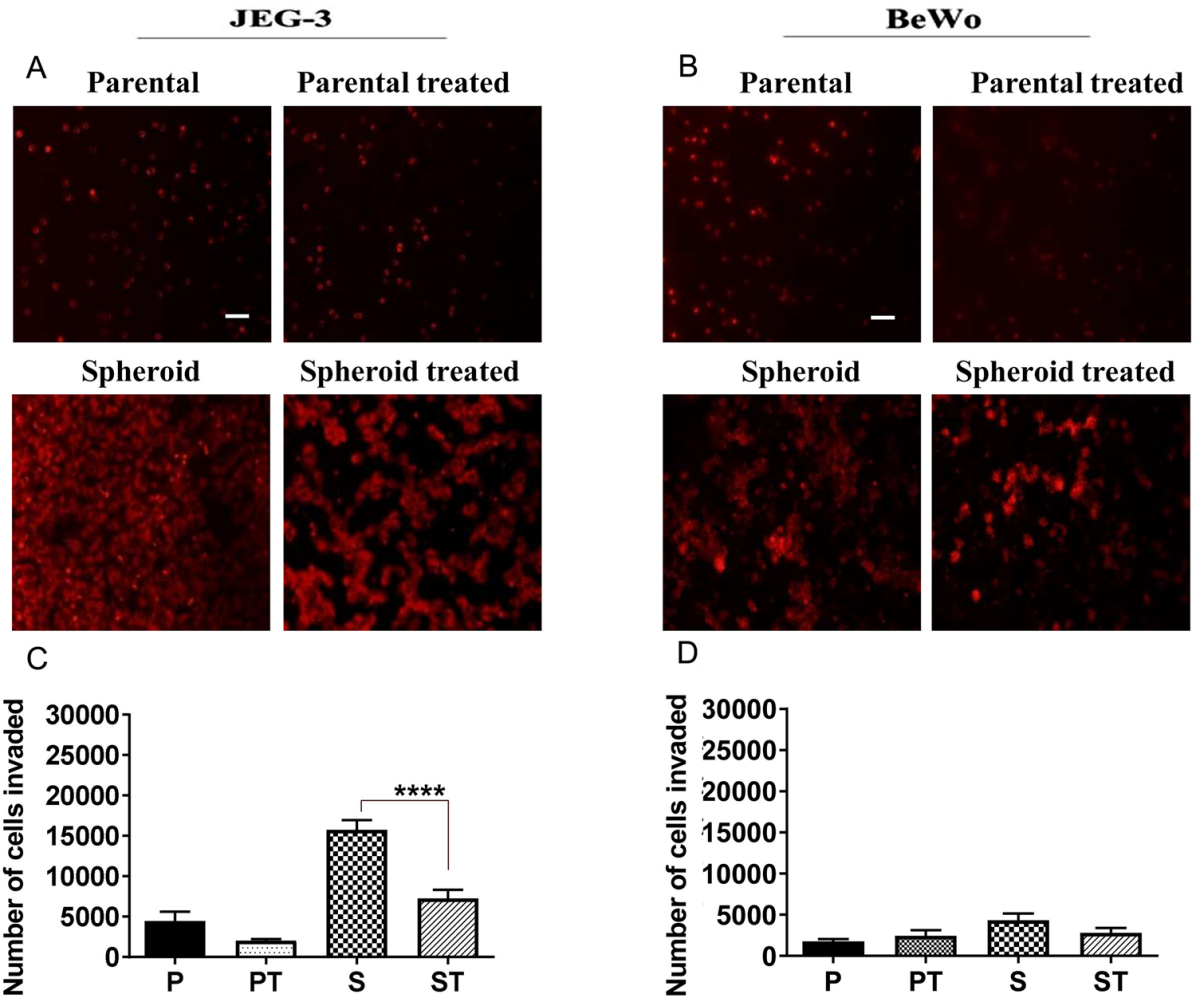
Comparative cell invasion of choriocarcinoma cell lines. Panels **A** and **B**: Represent the number of JEG-3 and BeWo cells invaded at 24 h. Image was taken of the bottom coated membrane using an Olympus fluorescence microscope. Objective magnification 20X (Scale bar = 100 μm). Panels **C** and **D**: Represent the quantitative analysis of the number of cells invaded using ImageJ and GraphPad software. Statistical significance was determined using a t test. Data represent the mean ± SED of three individual experiments, each performed in triplicate (****p < 0.0001)

We also investigated the effects of doxorubicin on the invasive capacity of spheroidal cells originating from choriocarcinoma cell lines, using BD Bio-Coat tumour invasion assay kit. The number of cells invaded by untreated spheroids was found to be higher than that of doxorubicin-treated spheroids of both cell lines [see Fig. 5]. This was found to be significant in JEG-3 untreated spheroids [see Fig. 5; panel **A** (left)]. Also, spheroidal cells produced from these choriocarcinoma cells showed higher invasion potential than their parental cells. However, the number of cells invaded in the BeWo cell line was generally lower than JEG3.

**Fig. 6:**
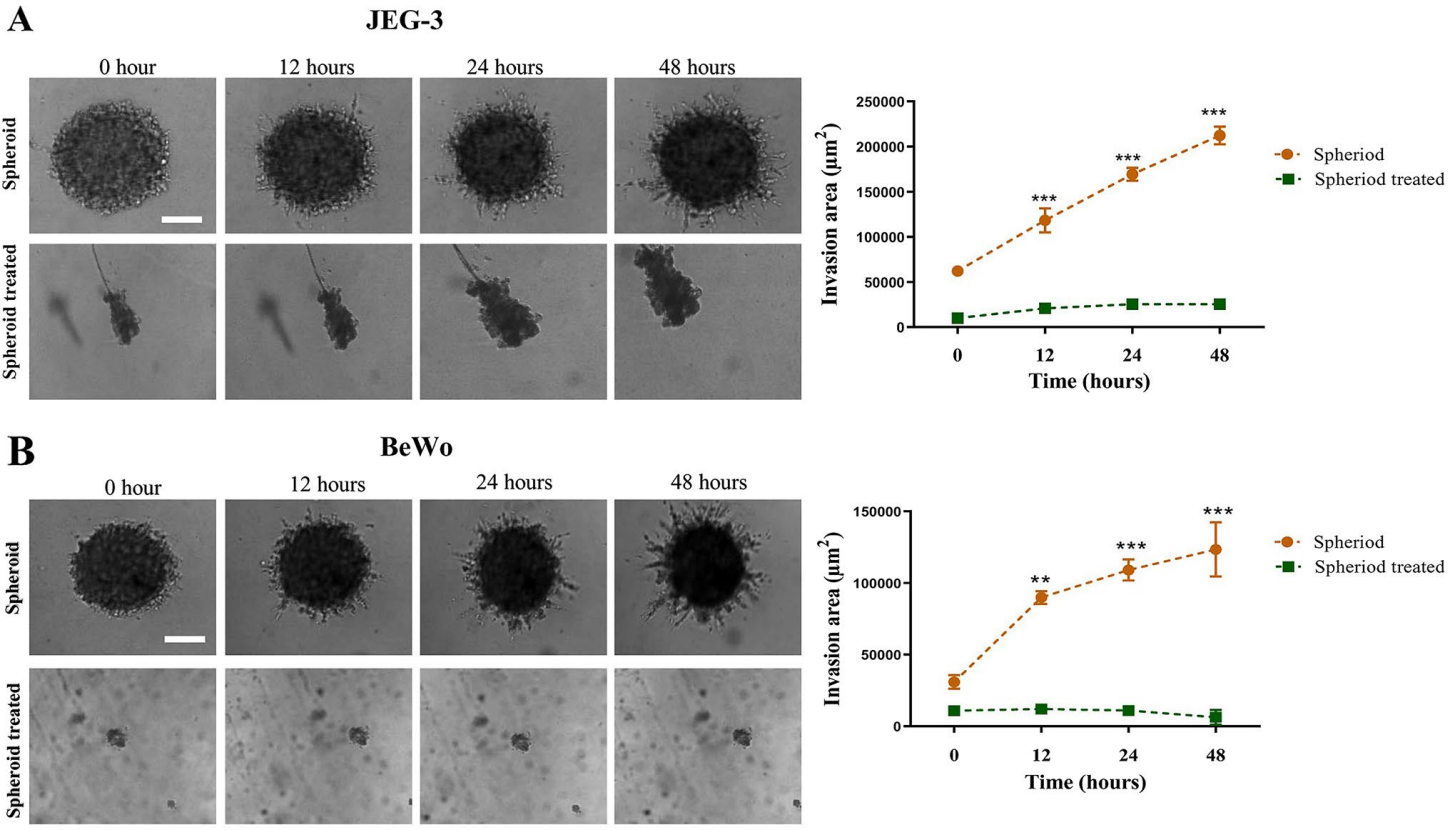
3D Invasion of untreated and doxorubicin-treated spheroids of choriocarcinoma cell lines. Invasion pattern of untreated and treated JEG-3 and BeWo spheroids and corresponding quantitative analysis of invasion areas are shown in panels **A** and **B**, respectively. A two-way ANOVA followed by Sidak’s for multiple comparisons was carried out. Data represent the mean ± SEM of three individual experiments, each performed in quintuplicate ***p < 0.001; **p < 0.01). Objective magnification = 10X

To further confirm these results, and to confirm that the spheroids themselves (without dispersing into single cells) retain the invasion capacity, we used the 3D spheroid BME cell invasion assays to compare the invasive potential of untreated and doxorubicin-treated spheroidal cells. In both choriocarcinoma cell lines, the size of the untreated spheroids was found to be larger than doxorubicin-treated spheroids (Fig. 6, A and B). In the case of JEG-3 cells, there was a significant increase in invasion potential observed in untreated spheroids compared to doxorubicin-treated spheroids at 12, 24 and 48 h. Similarly, in BeWo cells, the untreated spheroids showed a higher invasion potential than doxorubicin-treated spheroids at 12, 24 and 48 h. However, the doxorubicin-treated spheroids from both cell lines only produced a collection of spheroid-like bodies without any invasive properties. It could be observed from Fig. 6 that doxorubicin-treated spheroids did not show any signs of invasion even after 48 h, after which even the size started decreasing.

## Discussion

The emergence of self-regenerating heterogeneous groups of cancer stem cells has been hindering the long-term efficacy of many chemotherapeutic approaches. In fact, this is a selective pathophysiological adaptation of tumour cells in response to the drug itself (Czerwińska et al. 2018; Hepburn et al. 2019). Therefore, in vitro models to study the effects of chemotherapy on the production of CSC’s are warranted. We have previously shown that the transformed first trimester trophoblast cell lines were able to produce self-renewing pools of stem-like spheroidal cells (Balahmar et al. 2018). In this study, we checked the ability of resistant choriocarcinoma cells to produce spheroids, to express stem cell markers and to invade.

Choriocarcinoma is a type of gestational trophoblastic disease (GTD) which usually arises due to uncontrolled proliferation of trophoblastic cells (Smith et al. 2005). Studying choriocarcinoma is important because they are malignant and can cause secondary tumours in the liver, lungs and in very few cases brain (Naveen Kumar et al. 2019). Cancer cells have the ability to form 3D spheroids, which have been used to study pathogenesis (Bapat et al. 2005) and to determine the effect of drugs (Bapat et al. 2005) mimicking the pathological condition. The expression profiles of these 3D cultures have been found to more accurately mimic the physiological expression than the 2D cultures (Hirschhaeuser et al. 2010). The spheroids exhibit several properties of the solid tumours as they exhibit ECM secretion, cell to cell contact and other physiological features similar to the solid tumours (Rodrigues et al. 2018). The 3D spheroid model responds to cues based on stimuli such as multi-layered permeability barrier, hypoxic levels, proliferation zones, tumour heterogeneity and further similarities, which makes them a suitable model for studying the pathology and efficacy of drug under in vitro conditions (Sant and Johnston 2017).

In this study, JEG-3 and BeWo cells were used as a model system representing trophoblast tumours. We initially checked the ability of the cells to form 3D spheroids. Further the cell lines were treated with one of the common chemotherapeutic agents, doxorubicin, to investigate the abilities of these cells to survive and produce spheroids as a response to chemotherapy. When both cell lines were allowed to grow in ultra-low attachment, they produced spheroids. Moreover, spheroids generated from doxorubicin-resistant cells expressed various stem cell markers. Trophoblast cells have been reported to form spheroids in vitro by several authors (including our team) (Baal et al. 2009; Gonzalez et al. 2011; Balahmar et al. 2018). Spheroids are laden with cells that exhibit characteristics of stem cells (Bapat et al. 2005). Hence, we studied the status of various stem cell markers, such as *NANOG, OCT4* and *SOX2* in the drug-treated and non-treated spheroids of these two cell lines. These stem cell markers are the essential transcription factors for the maintenance of pluripotent embryonic stem cells. Previous reports show that these factors are also activated in tumour cells and the status of tumour aggressiveness is directly related with the expression of these factors (Gwak et al. 2017). In our study, the expression of *Sox2* and *Nanog* was clearly observed in both the cell lines. However, the parental cells of JEG-3 did not show the expression of *Oct4*. Looijenga and Oosterhuis (2013) showed that some of the teratoma tumour cells (including choriocarcinoma cells) did not express *Oct4*. However, parental cells (untreated and treated) of BeWo exhibited immunofluorescence for *Oct4*. In the case of spheroids, *Oct4* (together with other stem cell markers) expression is observed in both cell lines. This shows that these cell lines have the ability to undergo different forms of transformation in the expression of stem cell markers during spheroid generation.

The immunoblotting data suggest the expression of these stemness markers that are generally relatively higher in spheroidal cells compared to parental cells. In general, there is an increased expression of *Nanog*, *Oct4* and *Sox2* in the spheroids when compared with the parental cells. Since these three markers are termed as master regulators of stemness in the cell (Hepburn et al. 2019), it can be inferred that in spheroids the trophoblast cells and the choriocarcinoma cells undergo transition to form stem-like cells. Then, it was essential to ascertain that these CSC spheroidal cells are capable of invasion.

The data from 2D invasion suggested that doxorubicin-treated spheroids exhibited less invasion potential than the untreated spheroids. Overall, it is clear that the spheroids produced from both transformed trophoblast and choriocarcinoma cell lines were capable of 2D invasion. However, the invasive potential of spheroids will be better studied in 3D invasion systems (Vinci et al. 2015). Especially the fact that we wanted to compare the invasion potential of untreated and doxorubicin-treated cells. We wanted to check whether the spheroids per se (i.e. without dispersing into single cells) have the ability to invade. Untreated spheroids of both choriocarcinoma cell lines showed a distinct increase in the invading area with time. These data confirm that spheroids derived from untreated cells can retain, if not increase the potential to invade. However, doxorubicin-treated spheroids from both JEG-3 and BeWo cells have lost the ability to invade. The spheroid became distorted and did not show any invasion in either of the cell lines. This may be due to the persisting effect of the doxorubicin treatment in these cell lines.

The expression of *NANOG*, *OCT4* and *SOX2* is necessary for maintenance of stemness in various stem cells and are considered as master regulators of pluripotency (Leis et al. 2012; Hepburn et al. 2019). According to immunoblot data, spheroids have significantly higher expression of *Nanog, Oct4* and *Sox2* than the parental cells. Small changes in the expression of these three factors have been shown to produce different lineages in human embryonic stem cells (Wang et al. 2012). Kallas et al., (2014) showed that the regulation of *SOX2* expression is essential for self-renewal in the population of embryonic stem cells. When the expression of *Sox2* is decreased, cells start differentiating. In gastric cancer, higher *Sox2* expression positively correlated with doxorubicin resistance and when *SOX2* was inhibited using siRNA, the ability of the spheroid formation was significantly affected. This also made cells of spheroids more vulnerable to doxorubicin and reduced tumorigenicity in vivo (Tian et al. 2012). Therefore, the higher expression of *SOX2* in resistant spheroids may confer the cells with doxorubicin resistance and ability to form spheroids. Apart from the development of stem cells/stem like cells, several other phenomenon have been put forward for the development of doxorubicin resistance including ABC transporters, activation of survival pathways, affecting epithelial-mesenchymal transition, besides mutations induced due to DNA-adduct formations (Cox and Weinman 2016; Micallef and Baron 2020). Our data also showed, in addition to doxorubicin resistance, that there was reduced invasion from drug-treated spheroids. This may be due to the persistent effect of doxorubicin on proliferation and invasion of the cells. Although spheroids exhibited reduced invasion, the stemness factors expression, especially *SOX2* which is associated with promoting the invasion of cancer and aggressiveness of cancer warrants further studies to check whether the stemness factor revives the invasion of the spheroids on further prolonged exposure.

## Conclusion

The spheroidal cells produced from both choriocarcinoma cells showed increased expression of the stem cell markers and enhanced invasion. The doxorubicin-resistant cells also exhibited the stem cell markers with reduced 3D invasion; further studies are warranted to study the nature of the stem-like cells and their suitability to further reveal how the trophoblast invasion is altered during the transformation into choriocarcinoma.

## Supplementary Information

Below is the link to the electronic supplementary material.

Supplementary file1 (DOCX 413 KB)
