# Supplementary material for: Doxorubicin resistant choriocarcinoma cell line derived spheroidal cells exhibit stem cell markers but reduced invasion
Source: 3 Biotech. 2022 Jul 20;12(9):184. doi: 10.1007/s13205-022-03243-x (PMC9300786; doi:10.1007/s13205-022-03243-x)
Supplement: Supplementary file 1 — Supplementary file1 (DOCX 413 KB) [file 13205_2022_3243_MOESM1_ESM.docx]

**
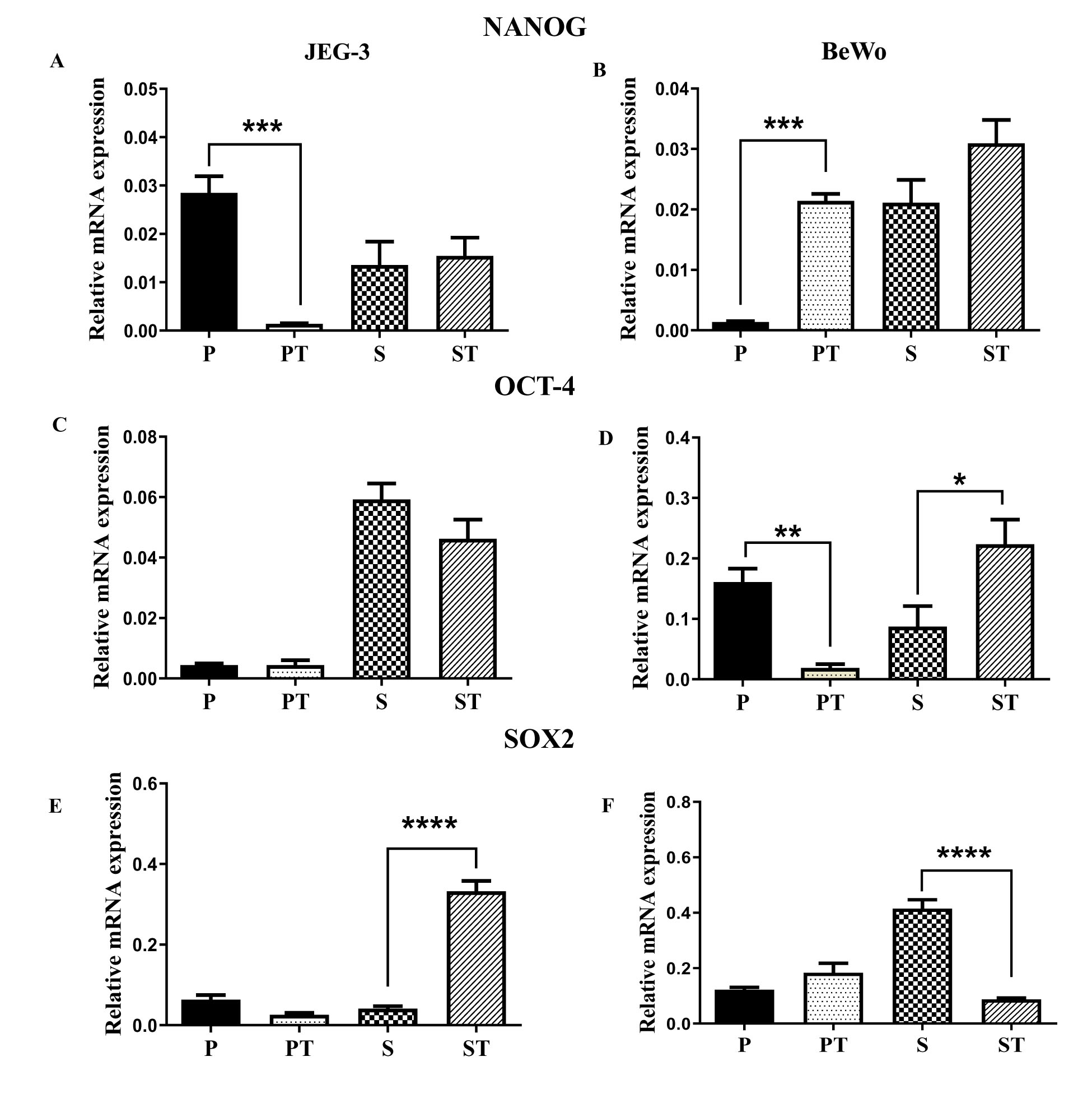
**

***Figure S1: Relative mRNA expression of NANOG, OCT4 and SOX2 in choriocarcinoma cells under different culture conditions.*** *The data for (i) NANOG expression are given in panels A and B; (ii) OCT4 expression are presented in panels C and D; (iii) SOX2 expression in panels E and F . Statistical significance was determined using a one-way ANOVA followed by Tukey's test for multiple comparisons. Data represent the mean ±SEM of three individual experiments, each performed in triplicate (****p<0.0001; ***p<0.001,**p<0.01; *p<0.05);* ***P****-Parental;* ***PT****-Parental treat;* ***S****-Spheroid;****ST****-Spheroid treat*
